# RNA targets of TDP-43: Which one is more important in neurodegeneration?

**DOI:** 10.1186/s40035-021-00268-9

**Published:** 2022-02-25

**Authors:** Dilara Halim, Fen-Biao Gao

**Affiliations:** Department of Neurology, UMass Chan Medical School, Worcester, MA 01605 USA

Nuclear loss and cytoplasmic aggregation of TDP-43 is the most common shared pathological feature of frontotemporal dementia (FTD) and amyotrophic lateral sclerosis (ALS), a motor neuron disease also known as Lou Gehrig's disease [1]. TDP-43 pathology is found in 97% of ALS cases, approximately 45% of FTD cases, and in some other neurodegenerative diseases such as Alzheimer’s disease [2]. However, the direct mechanisms by which TDP-43 dysregulation contributes to neurodegeneration remain largely elusive. TDP-43 is an RNA-binding protein with multiple roles in RNA metabolism, including transcription, splicing, transport, and the localization and stability of its target mRNAs, as well as in microRNA biogenesis [3]. TDP-43 has thousands of RNA targets, but it is largely unknown which targets, if any, directly contribute to disease. Nor is it known whether any of them can be either manipulated as therapeutic targets to influence disease progression or, if not, could serve as biomarkers of TDP-43 pathology.

A key nuclear function of TDP-43 is to regulate alternative splicing, most notably to suppress the inclusion of cryptic exons, intronic sequences that are normally not spliced into mature mRNAs [4]. This dysregulation may result in the inclusion of a new stretch of amino acids in the protein, the production of a truncated protein, or a loss of the full-length protein through nonsense-mediated decay (NMD) of the mRNAs in which premature termination codons (PTCs) are introduced by cryptic exons (Fig. 1). RNA-seq analysis of human neurons with reduced TDP-43 expression revealed that one of the most downregulated genes is stathmin 2 (*STMN2*), encoding an axonal growth-associated microtubule-stabilizing protein expressed only in neurons [5, 6]. This downregulation is specific to human *STMN2* and is not conserved in rodents [5, 6]. The extent to which the loss of full-length STMN2 function contributes to ALS/FTD pathogenesis and clinical phenotypes is still not known. Nonetheless, restoring expression of full-length STMN2 represents a novel potential therapeutic approach.

**Fig. 1:**
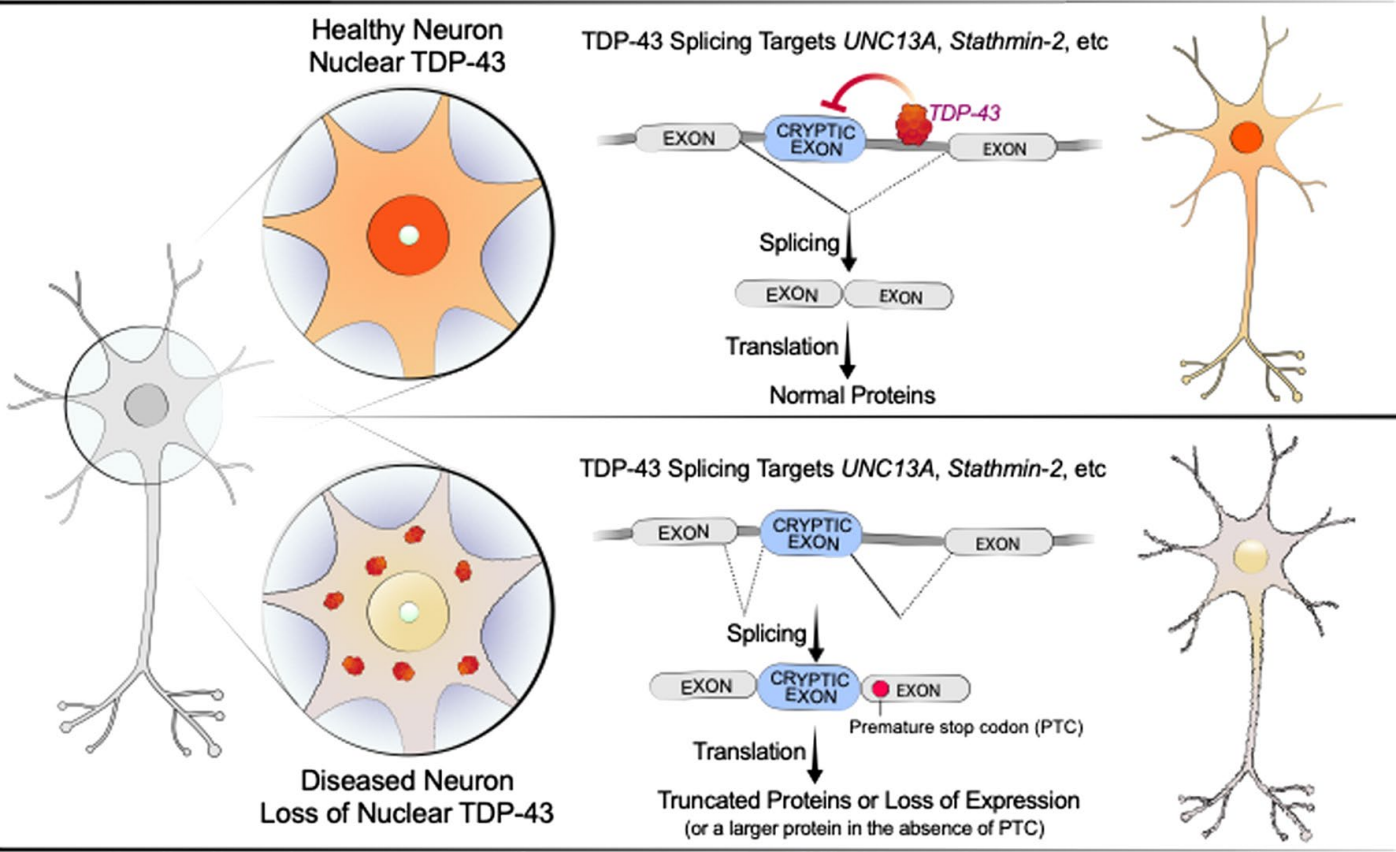
Suppression of cryptic exon splicing by TDP-43. One of the normal functions of TDP-43 in the nucleus is to suppress the splicing of cryptic exons located in introns. Nuclear depletion of TDP-43 results in the inclusion of these cryptic exons in mature mRNAs, which leads to the production of truncated proteins (as in the case of STMN2 due to an alternative polyA site in the cryptic exon) or loss of normal expression (as in the case of UNC13A due to the presence of a premature termination codon (PTC) and subsequent nonsense-mediated decay of its mRNA)

In studies to identify other key functional targets of TDP-43 that may directly contribute to disease pathogenesis, Ma et al. [7] and Brown et al. [8] identified novel cryptic exons regulated by TDP-43, including one in *UNC13A* (Fig. 1). Ma et al. [7] reanalyzed a published RNA-seq dataset for abnormal splicing events. This dataset was obtained after separating TDP-43-positive and TDP-43-negative neuronal nuclei in ALS/FTD patient postmortem brains to identify transcriptomic changes caused by nuclear TDP-43 depletion. Similarly, Brown et al. did an RNA-seq analysis of human neurons derived from induced pluripotent stem cells (iPSCs) after depletion of TDP-43 by CRISPR inhibition [8]. Among dozens of novel cryptic exons, both groups identified *UNC13A* as one of the most robust mis-spliced genes. Brown et al. also detected a mis-splicing event in another UNC13 family member, *UNC13B* [8].

To confirm their findings, Ma et al. and Brown et al. used human neuronal cell lines and iPSC-derived neurons to reduce TDP-43 levels. They found that TDP-43 depletion caused inclusion of the cryptic exon in the *UNC13A* transcript, confirming a role for TDP-43 in suppressing cryptic exons. This effect seems to be direct, as TDP-43 has a binding site within the intron containing the cryptic exon in *UNC13A* mRNA [7, 8]. Moreover, they found that the *UNC13A* RNA containing the cryptic exon and a PTC is a degradation target of NMD, resulting in reduced levels of *UNC13A* mRNA and protein. This effect is distinct from that of aberrant *STMN2* mRNA, which does not undergo NMD after cycloheximide treatment but is instead translated into a truncated STMN2 due to a predicted alternative polyA site in the cryptic exon [8].

Next, Ma et al. and Brown et al. analyzed by bulk RNA sequencing a large set of brain samples from FTD or ALS patients with TDP-43 pathology (thus named as FTD-TDP and ALS-TDP) and without TDP-43 pathology, including FTD-FUS, FTD-TAU, and ALS-SOD1 (named based on the presence of FUS, TAU, or SOD1 pathology). The *UNC13A* cryptic exon was found in samples from patients with TDP-43 pathology but not in those from controls or patients without TDP-43 pathology. Thus, the *UNC13A* cryptic exon is a specific feature of TDP-43 proteinopathies. The researchers also performed *in situ* hybridization with probes specific to the cryptic splicing site in *UNC13A* mRNA. The *UNC13A* cryptic exon-containing RNA was detected in neuronal nuclei with TDP-43 depletion in FTD-TDP patient brains but not in neurons with nuclear TDP-43 in patient samples or in controls. Both groups also found a correlation between the levels of the *UNC13A* cryptic exon and phosphorylated TDP-43 in patients [7, 8]. These results provide further evidence for a direct link between TDP-43 pathology and the *UNC13A* cryptic exon and raise the possibility that this exon could be developed into another biomarker for the majority of FTD and ALS cases.

*UNC13A* mRNA is a very interesting target of TDP-43 because it was previously identified as a risk factor for sporadic ALS and ALS/FTD through genome-wide association studies (GWAS) [9]. Since UNC13A participates in vesicle maturation and neurotransmitter release [10], loss of its activity may compromise neuronal function in patients. Both groups found that the two SNPs most strongly associated with disease are located either within the *UNC13A* cryptic exon or in the intron near the cryptic exon. In addition, Ma et al. identified a novel variant, also located in the intron that contains the cryptic exon, as a risk factor for disease. Both groups showed a strong correlation between the disease-associated SNPs and the abundance of the cryptic exon in patient brains. Expression of minigenes containing these disease risk variants in cultured cells established a direct effect of these variants in increasing cryptic exon inclusion in TDP-43-depleted cells. These findings support the notion that some disease risk variants promote inclusion of the cryptic exon upon loss of nuclear TDP-43, providing mechanistic insight into why these variants are associated with increased risk for ALS and ALS/FTD and further highlighting the key role of TDP-43 in disease pathogenesis [7, 8]. Because of this genetic link between *UNC13A* and disease, blocking the splicing of the cryptic exon in *UNC13A* is another promising therapeutic approach.

Several issues remain to be addressed. Since only a small percentage of neurons in patient brains show loss of nuclear TDP-43 at the end of disease, the extent to which the *UNC13A* cryptic exon contributes to disease needs to be further established. Moreover, through its interactions with thousands of target mRNAs, TDP-43 affects many aspects of RNA metabolism. Thus, it remains to be seen whether correcting the expression level of one target mRNA is sufficient to offer tangible clinical benefits while many other downstream pathways remain dysfunctional. Simultaneous blocking of cryptic exon splicing in multiple key target mRNAs of TDP-43 may offer a better chance of success. Nonetheless, the GWAS results strongly suggest that the contribution of *UNC13A* missplicing to disease pathogenesis may in fact be tangible, and worth pursuing. Thus, these two new studies greatly enrich our understanding of key functions of TDP-43 and suggest another cryptic exon as a novel target for therapy and as a potential biomarker for both familial and sporadic ALS/FTD.

## Data Availability

Not applicable.

## Abbreviations

ALS: Amyotrophic lateral sclerosis
FTD: Frontotemporal dementia
GWAS: Genome-wide association studies
iPSCs: Induced pluripotent stem cells
NMD: Nonsense-mediated decay
PTC: Premature termination codons
